# Investigating Helmet Promotion for Cyclists: Results from a Randomised Study with Observation of Behaviour, Using a Semi-Automatic Video System

**DOI:** 10.1371/journal.pone.0031651

**Published:** 2012-02-15

**Authors:** Aymery Constant, Antoine Messiah, Marie-Line Felonneau, Emmanuel Lagarde

**Affiliations:** 1 INSERM U897-IFR99, Equipe Avenir Prévention et Prise en Charge des Traumatismes, ISPED, Bordeaux, France; 2 EHESP School of Public Health, Rennes, France; 3 Social Psychology Department, Université Victor Segalen Bordeaux 2, Bordeaux, France; Research and Development Corporation, United States of America

## Abstract

**Introduction:**

Half of fatal injuries among bicyclists are head injuries. While helmet use is likely to provide protection, their use often remains rare. We assessed the influence of strategies for promotion of helmet use with direct observation of behaviour by a semi-automatic video system.

**Methods:**

We performed a single-centre randomised controlled study, with 4 balanced randomisation groups. Participants were non-helmet users, aged 18–75 years, recruited at a loan facility in the city of Bordeaux, France. After completing a questionnaire investigating their attitudes towards road safety and helmet use, participants were randomly assigned to three groups with the provision of “helmet only”, “helmet and information” or “information only”, and to a fourth control group. Bikes were labelled with a colour code designed to enable observation of helmet use by participants while cycling, using a 7-spot semi-automatic video system located in the city. A total of 1557 participants were included in the study.

**Results:**

Between October 15th 2009 and September 28th 2010, 2621 cyclists' movements, made by 587 participants, were captured by the video system. Participants seen at least once with a helmet amounted to 6.6% of all observed participants, with higher rates in the two groups that received a helmet at baseline. The likelihood of observed helmet use was significantly increased among participants of the “helmet only” group (OR = 7.73 [2.09–28.5]) and this impact faded within six months following the intervention. No effect of information delivery was found.

**Conclusion:**

Providing a helmet may be of value, but will not be sufficient to achieve high rates of helmet wearing among adult cyclists. Integrated and repeated prevention programmes will be needed, including free provision of helmets, but also information on the protective effect of helmets and strategies to increase peer and parental pressure.

## Introduction

In its latest global assessment of road safety, the World Health Organization (WHO) reported that half of the 1.2 million fatalities occurring each year on the world's roads concern vulnerable road users (VRUs), with children and the elderly being overrepresented among victims [1]. Pedestrians, pedal cyclists, and motor cyclists are considered as vulnerable since they benefit from little or no external protective devices that would absorb energy in a collision. They constitute with almost no exception the weak party in a road traffic crash.

Beyond preventive policies such as traffic-calming measures, enforcement of drunk driving legislation and increased visibility/conspicuity [1], individual passive safety is also essential to protect VRUs from injury. As far as motorised two-wheelers are concerned, the most effective protection that can be offered is the helmet. Evidence from a systematic review shows that it reduces the risk of fatal injuries by 42% [2]. When it comes to cyclists, authors of a Cochrane Collaboration analysed five case-control studies and concluded that helmets reduce by 63% to 88% the risk of head, brain, and severe injuries among cyclists. They concluded that bicycle riders of all ages should be encouraged to wear helmets [3]. According to the European Transport Safety Council [4], the death risk per 100 million person kilometres travelled is 5.4 for bicyclists in Europe, compared with 0.7 for car users and 0.07 for bus and coach passengers. Because about half of fatal and serious injuries among bicyclists are head injuries, helmet use is recommended [3] and sometimes even compulsory in a small number of countries.

There is, however, still no consensus on the best policy regarding helmets for bicyclists. First, the accuracy of evaluating studies is debated. When sources of bias are controlled for, the protective effects attributed to bicycle helmets [5] become smaller than originally estimated [6]. Second, it has been argued that bicyclists' helmet use has the potential to make other drivers less cautious concerning cyclists [7] and to increase cyclists' propensity to take more risks (a phenomenon called risk compensation or risk homeostasis) [8]. Both hypotheses, however, rely upon little observational evidence. Finally, there is also a debate on whether helmet use should be compulsory [9]. Some authors state that a helmet law might deter people from cycling, and thus diminish the benefits to health of regular exercise provided by cycling, [10], [11]. Such a decrease in cycling may also increase cyclists' vulnerability due to the lower awareness of this population by other road users [12].

Effective strategies to promote bicyclist helmet use are therefore still to be identified. These may include health education programmes, subsidised or free helmet distribution programmes, media campaigns, or interventions that include elements of the above. Community-based interventions and those providing free helmets have an effect on reported use, but were mostly aimed at children [13].

Identifying successful interventions should also provide an opportunity to understand the determinants of such protective behaviour. Psychosocial models such as the Theory of Planned Behaviour (TPB) [14], [15], the Locus of Control, and the Health Belief Model [16] provide a theoretical framework that has frequently been applied to study the determinants of behavioural intention. Among these models, TPB is of particular interest since all its components were shown to correlate with the intention to use a helmet among adolescents [17]. These are attitudes toward the behaviour (affective and instrumental evaluations of performing the behaviour), subjective norms (perceived social pressure to perform a behaviour or not), and perceived behavioural control (the perceived ease or difficulty of performing the behaviour). Available studies suggest that the main determinant of helmet use is cyclists' motivation [18]. All such studies are, however, hampered by substantial uncertainty regarding the accuracy of self-reported behaviour, which is potentially subject to bias such as social desirability [19], [20] and recall bias. [21].

### Objectives

In the present study, we aimed to assess the influence of two strategies for promotion of helmet use, using a randomised controlled protocol, and with direct observation of behaviour by a semi-automatic video system. Factors influencing adoption of helmet use were elicited with analyses based on data from a questionnaire completed by participants at inclusion.

## Methods

### Trial design, settings and participants

We carried out a single-centre randomised controlled study, with four balanced randomised groups in Bordeaux, a city of 600,000 inhabitants located in the south-west of France. Participants were recruited from June 19th 2009 to August 13^th^ 2010 at a municipal centre (“La Maison du Vélo”) created to promote bicycle use. In this setting, people can borrow a bicycle for free for a minimum period of 4 months. Oral and written information about the study was delivered to all individuals entering the centre to borrow a bicycle. Only people declaring they were borrowing a bicycle for their own exclusive use were asked to participate. They were informed that their bicycle rides could be anonymously video-recorded during their daily trips. Two cinema tickets were offered as reward.

### Interventions

Participants were divided into four groups using the day of the visit as random assignment. Recruitment took place five days a week, from Monday to Friday. The first four days of the study were randomly assigned to one of the four groups (information, helmet, helmet and information, control), and the same pattern was repeated throughout the study period. Participants in experimental arm #1 received a brochure designed by the French National Institute for Prevention and Health Education (INPES), which promotes bicycle helmet use. The brochure contains recommendations on how to wear a helmet properly, and statistics on head injury risk reduction by helmet use. Standardised oral information was given together with the brochure. Participants in experimental arm #2 received a helmet for free; and participants in experimental arm #3 received both the brochure and a free helmet.

Sociodemographic variables (gender, age, education level, professional activity), history of bicycle injuries (caused by falls and collisions) in the last 12 months, and helmet use in the past month were collected at inclusion through a standardised questionnaire. Several psychological variables from the TPB model were assessed on a 10-point scale: a helmet's value in protecting the head and face (instrumental attitude: 1 = absent; 10 = extremely high) and agreement with statements related to affective attitudes towards helmet: “helmet makes people ridiculous” or “old-fashioned” (1 = totally disagree; 10 = totally agree). The descriptive norm was measured by asking respondents to assess the percentage of helmet use among cyclists of the same age and gender. Peer pressure was measured by asking participants whether close family members and friends encouraged them to wear a helmet on a four-point scale (1 = certainly not; 2 = no; 3 = yes, 4 = certainly yes). Participants were also asked to rate their perceived risk of bicycle injury (1 = absent; 10 = extremely high), and their level of competence and cautiousness while riding, using a 10-point scale (1 = very low; 10 = very high). Personal data (name, date of birth, address) were not collected. Each participant was given a unique identification (ID) study number. A corresponding coloured ID code was put on each bicycle rear mudguard to ensure participant ID number identification during in-field observations.

### Outcome

**Figure 1:**
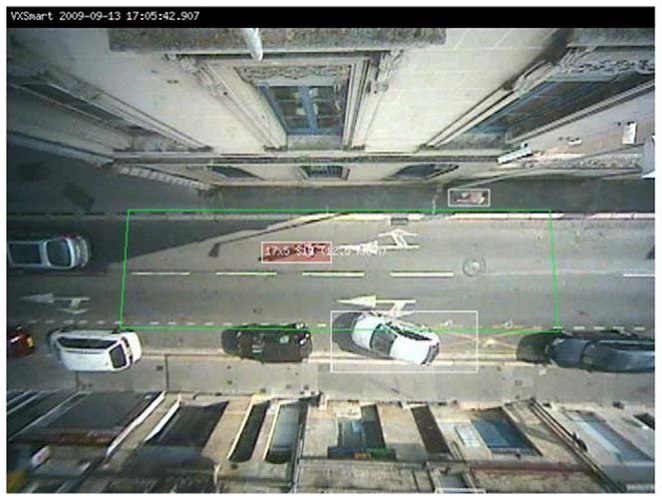
Capture of a cyclist's movements by a camera from a vertical angle.

**Figure 2:**
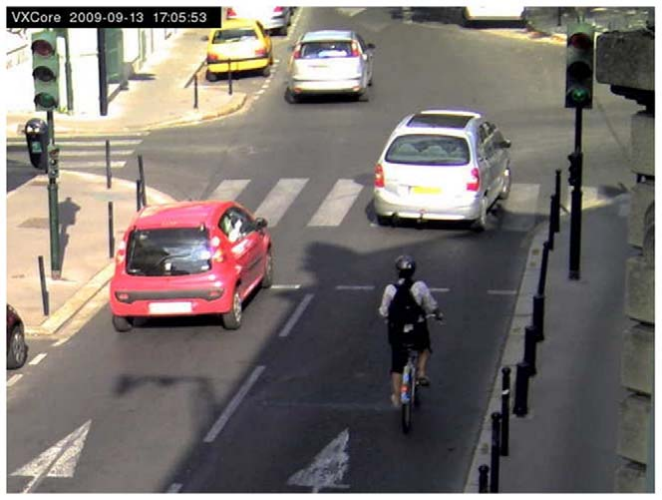
Picture of detected cyclist from behind at a 45 degree angle.

Five observation sites were deployed in the urban centre of Bordeaux. Two of them made observations in both directions, leading to a total of seven observation spots. Sites were selected according to the following criteria: (i) available electrical power and either internet or phone line access; (ii) a road environment compatible with bicycle traffic; (iii) a range of different street configurations: cross-roads with traffic lights, one-way and two-way roads, traffic mixing including buses, trams and pedestrians. Sites were connected to the main server in our settings via the internet. Each observation spot comprised two cameras. A first camera with a built-in image analysis processor recorded cyclists from above by forming a 90 degree vertical angle with the road surface. It was programmed to detect moving objects, isolate cyclists, and calculate speed (Figure 1). A second synchronised high-definition camera automatically took a photo of each detected cyclist from behind at a 45 degree angle (Figure 2). This photo was subsequently used manually to perform complementary measurements such as helmet use, reflector use, several types of infringements, and ID code reading. All cameras were installed on municipal buildings and collected data six hours a day seven days a week. A trained video coder visualised each picture recorded on the central server and entered the corresponding data in a database.

### Blinding

To ensure confidentiality, participants' informed consents were kept away from survey material in a locked box. Inclusion questionnaires were checked at the end of each interview. Anonymous study numbers and corresponding colour codes were used for participant individual identification and follow-up. Quality of video coding was assessed during a four-week preliminary phase of the study. The colour code given to each cyclist did not indicate the arm allocation. The staff in charge of coding in-field data from video records were blind to the randomisation group and to other information collected by questionnaire. In addition, video and image records were stored for a maximum of one week. Records were systematically discarded after data coding. A preliminary study was performed between May 10^th^ 2009 and June 15^th^ 2009 to measure the performance of the automatic video detection system. Counts of cyclists' movements produced by the system were compared with manual coding. Results showed that 2390 out of 3583 cyclists' movements were detected and recorded, achieving a 67% detection rate.

### Statistical methods

Baseline characteristics were compared between randomisation groups and excluded participants using the Chi-square test for nominal data and Kruskal–Wallis one-way analysis of variance for ordinal data. The colour code set on the bicycle mudguard was used to match data collected on actual helmet use with data on the participant randomisation group and inclusion questionnaire. Participants who were not seen by any of the cameras were not included in the current analysis. Chi-square tests were conducted with each participant as a statistical unit to compare baseline characteristics and helmet use (observed at least once) between randomisation groups. Because each participant could be observed several times during the study period, a second analysis was performed, using every movement made by the study participant and recorded by camera as statistical units. The Generalised Estimating Equation (GEE) technique extends the generalised linear model to include analysis of repeated measurements or other correlated observations [22], [23]. We used autoregressive logistic regression as it allows modelling of the binary outcome variable (observed helmet use) with repeated observations at different time periods for the same individual [24]. With this method, the efficacy of each promotional strategy was assessed by comparing repeated behavioural observations between randomisation groups. Factors influencing helmet use were assessed, based on data related to age, gender, perceptions, beliefs, and peer pressure as collected during the inclusion interview. Estimates (Model 1) were expressed as odds ratio with 95% confidence intervals adjusted for helmet provison (OR [95% CI]). Significant estimates from Model 1 were analysed in a multivariate model (Model 2) and their interactions were investigated in a third model. Statistical analyses were performed using the SPSS statistical package, version 16 (SPSS, Chicago, Illinois, United States).

## Results

### Participant flow and recruitment

It was not possible to enumerate all potential participants entering the centre to borrow a bicycle, since during busy times the call for participation was made using an information sign on the centre's main desk. During the study period, however, around 6000 loan agreements were concluded and 1798 participants agreed to participate in the study (estimated recruitment rate: 30%). Participants who reported previous helmet use (N = 241) were excluded from analyses.

### Baseline data

The characteristics of the 1557 study participants in the four randomisation groups are reported in Table 1, and compared with the characteristics of the 241 participants who were excluded. No between-group imbalance was found regarding gender, age, education, occupation and history of bicycle incidents. Previous helmet users (excluded participants) reported higher scores of subjective and descriptive norms, and lower scores of negative attitudes towards helmet use than included participants (p<0.001). Reports showed that participants considered themselves as skilled and cautious riders, with a limited risk of being injured while riding. Helmet was considered as effective in preventing head injury, but not facial injury. On average, they reported little encouragement from close friends and family to wear a helmet.

**Table 1:** Characteristics of included and excluded participants.

	Control group	Information only	Helmet only	Information and helmet	Excluded*
	N = 382	N = 410	N = 382	N = 383	N = 241
Sociodemographic characteristics N (%)‡					
Female gender	214 (56.0)	240 (58.5)	223 (58.4)	231 (60.3)	134 (55.6)
Mean age†	31.1 (11.5)	31.7 (12.2)	31.1 (12.0)	32.0 (11.5)	32.5 (12.6)
High school diploma	330 (86.8)	356 (86.8)	343 (90.3)	331 (86.6)	206 (85.5)
Professionally active/student	326 (85.8)	338 (82.6)	319 (83.9)	320 (83.6)	203(84.2)
Bicycle incident in the last 12 months	63 (16.5)	69 (16.9)	64 (16.8)	63 (16.5)	41 (17.0)
Perception of own behaviour (rated on a 10-point scale)†					
Self-reported caution while riding	7.5 (1.4)	7.5 (1.9)	7.4 (1.9)	7.5 (1.8)	7.8 (1.8)
Self-rated skill while riding	7.7 (1.5)	7.8 (1.6)	7.6 (1.7)	7.6 (1.6)	7.8 (1.6)
Perception related to risk and instrumental attitudes toward helmet (rated on a 10-point scale)†					
Risk of bicycle-related injury	4.3 (2.6)	4.1 (2.6)	4.0 (2.5)	4.2 (2.5)	4.2 (2.6)
Helmet efficacy in preventing head injury	8.6 (2.3)	8.5 (2.4)	8.5 (2.2)	8.9 (1.9)	8.9 (1.6)
Helmet efficacy in preventing face injury	5.4 (3.1)	5.6 (3.0)	5.7 (3.1)	5.8 (3.1)	6.0 (3.0)
Affective attitudes towards helmet (rated as a 10-point scale)†					
“helmets make people look ridiculous”	5.8 (3.2)	5.8 (3.2)	5.7 (3.2)	5.9 (3.1)	4.7 (3.1)
“helmets make people look old-fashioned”	5.0 (3.1)	5.4 (3.2)	5.3 (3.1)	5.4 (3.2)	4.2 (2.7)
Subjective norms (Rated on a 4-point scale)†					
**Close friend encouragement to wear helmet**	**1.5 (0.8)**	**1.5 (0.8)**	**1.5 (0.8)**	**1.5 (0.8)**	**2.3 (1.0)**
**Close family encouragement to wear helmet**	**1.9 (1.0)**	**1.9 (1.1)**	**2.0 (1.1)**	**1.9 (1.0)**	**2.9 (1.0)**
Descriptive norm (expressed as an estimated percentage)†					
Perceived bicycle helmet rate of use among peers	13.6 (12.3)	13.0 (12.8)	14.5 (12.4)	15.1 (13.2)	20.9 (16.8)
**Intention to wear a helmet in the near future**	**1.7 (0.7)**	**1.8 (0.8)**	**1.8 (0.8)**	**1.7 (0.7)**	**2.8 (0.8)**

‡ expressed as number (percentage). Between-group comparisons were made using Chi-square tests.

† expressed as mean score (standard deviation). Between-group comparisons were made using the Kruskal-Wallis non-parametric test. Significant results are marked in bold.

*241 participants excluded because they reported previous helmet use.

### Numbers analysed

Between October 15th 2009 and September 28th 2010, 2621 cyclists' movements, made by 587 participants, were captured by the system and considered as units of observations. Most observed participants were seen once (41.6%) or twice (19.6%).

### Outcomes

Participants seen at least once with a helmet amounted to 6.6% of all observed participants (Table 2), with higher rates in the two groups who received a helmet at baseline (helmet only: 11.4%; helmet and information: 9.2%) as compared with the others (control: 3.7%; information only: 3.2%; p = 0.008). Helmet use was observed in 3.8% (99 out of 2621) of observed movements, with higher rates in movements of participants who received a helmet at baseline (helmet only: 10.0%; helmet and information: 5.1%) as compared with the others (control: 1.1%; information only: 0.8%; p<0.001). Power calculation for equivalence tests shows that the study achieves a 78% power to test that the proportion of observed helmet use (at least once) in the information group (p = 5.9%) is equivalent to the proportion in the control group (p = 7.3%) with an error margin (delta) of +− 12%. GEE model estimates showed that the likelihood of observed helmet use was significantly increased among participants of the helmet only group (OR = 7.73 [2.09–28.5]) and among participants of the helmet and information group (OR = 4.33 [1.33–14.0]), compared with controls (Table 3). The likelihood of observed helmet use was similar in participants of the information only group and controls.

**Table 2:** Characteristics of observed participants (N = 587).

	Participants observed at least once		
	Total	Without helmet	At least once with helmet
Groups		N (%)	N (%)
All	587	548 (93.4)	39 (6.6)
Helmet and information	130	118 (90.8)	12 (9.2)
Helmet only	140	124 (88.6)	16 (11.4)
Information only	156	151 (96.8)	5 (3.2)
Control	161	155 (96.3)	6 (3.7)

**Table 3:** Number of observed movements, helmet use rate, and odds ratio with 95% confidence interval of observed helmet use.

Groups	Number of observed movements	Helmet use rate (%)	Odds ratio[95% confidence interval]†
Helmet and information	432	5.1	**4.33 [1.33–14.0]**
Helmet only	618	10.0	**7.73 [2.09–28.5]**
Information only	724	0.8	0.84 [0.23–3.02]
Control	847	1.1	1

† estimated by logistic regressions for autocorrelated data with application to repeated measures. Significant results are marked in bold.

### Ancillary analyses

A small number of participants were frequently observed by the system: one was seen 81 times, another 70, and a total of four were seem more than 40 times. These outliers explain the imbalance observed in the number of movements between randomisation groups. The impact of these outliers was assessed by restricting the number of movements per participant that were analysed, and by comparing new estimates with those computed without restriction. When limiting the number of movements to a maximum of 40, GEE model estimates varied only slightly (−0.89% of original values on average) and the number of movements became 618 in the helmet group and 432 in the helmet and information group, 626 in the information group and and 696 in the control group.

### Determinants of observed helmet use

**Figure 3:**
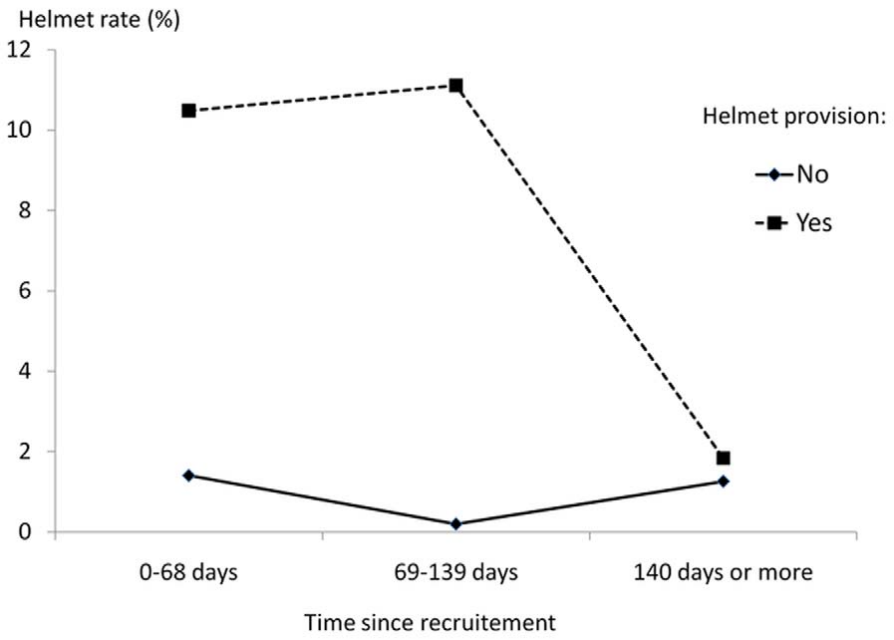
Helmet wearing rates in observed movements (N = 2621) as a function of time since recruitment and helmet provision at baseline.

The helmet only group and the information and helmet group were merged (and designated below as participants with helmet provision), showing that helmet provision had a significant effect on helmet use (OR = 6.55 [2.54–16.9]) as compared with participants of the other two groups. By contrast, the two groups with information were merged and compared with the other two groups, and no effect on helmet use was found (0R = 0.33 [0.26–1.58]). Therefore, we did not further investigate the effect of information. The benefit of helmet provision proved to be of limited duration: helmet use rate in observed movements decreased sharply after the fourth month following inclusion (Figure 3). Estimates adjusted for helmet provision showed that observed helmet use was associated with few variables from the TPB: with higher scores of encouragement by friends/family to wear a helmet and with reported helmet efficacy in preventing facial injury (Table 4), and with lower scores of perceived riding skills (model 1). In multivariate analysis, family encouragement to wear a helmet and reported efficacy in preventing facial inury remained significantly associated with observed helmet use (Model 2). Interactions between these variables and helmet provision were investigated with a 3-way model, but no term was significant, suggesting no differential impact of the intervention in these subgroups of participants with different pressure, perception and beliefs.

**Table 4:** Determinants of helmet use among participants who received a helmet at baseline.

	Model 1†	Model 2‡
Helmet provision		**5.31 [2.63–10.7]**
Male gender	0.78 [0.36–1.67]	
History of bicycle accidents	0.30 [0.07–1.25]	
Age (years)	0.98 [0.96–1.01]	
**ASSESSMENT OF OWN BEHAVIOUR**		
Self-reported caution while riding	0.97 [0.84–1.13]	
Self-rated skill while riding	**0.82 [0.69–0.96]**	0.86 [0.76–1.44]
**INSTRUMENTAL ATTITUDES TOWARDS HELMET**		
Perceived helmet efficacy in preventing head injury	0.62 [0.35–1.09]	
Perceived helmet efficacy in preventing face injury	**1.20 [1.09–1.33]**	**1.17 [1.05–1.30]**
**AFFECTIVE ATTITUDES TOWARDS HELMET**		
Agreement that “helmets make people look old-fashioned”	0.93 [0.84–1.03]	
Agreement that “helmets make people look ridiculous”	0.91 [0.84–1.00]	
**PEER PRESSURE**		
Close friend encouragement to wear helmet	**1.35 [1.01–1.81]**	1.05 [0.76–1.44]
Close family encouragement to wear helmet	**1.77 [1.38–2.27]**	**1.61 [1.23–2.09]**
Perceived bicycle helmet rate of use among peers (%)	1.00 [0.98–1.02]	

Statistical units are observed movements (N = 2621). Estimates and 95% confidence intervals were computed using logistic regressions for autocorrelated data with application to repeated measures.

## Discussion

Results from the 2621 cyclists' movements that were observed in the study showed that the provision of a free helmet convinced 6.6% of helmet non-users to wear a helmet. Provision of information using a leaflet designed to promote helmet use had no significant influence. The impact of the intervention faded within the first 5 months. Determinant analysis showed the influence of family encouragement and of the perceived efficacy of helmet protection against facial injury.

The main strengths of this study are (i) the comparison of groups that were randomised at inclusion, avoiding indication and selection bias, as the nature of the randomisation groups was revealed to the participants after the inclusion procedure. (ii) reliance on direct observation of helmet use enabling estimates not influenced by social desirability and other biases that would have occurred with self-reports. It is likely that rates of helmet use measured in previous studies based on self-reports are overestimated, which may lead to spurious conclusion regarding the efficacy of information campaigns. In our study, direct observation indicated very low rates of helmet use.

Previous studies based on self-reports have tried to identify barriers to helmet use [25], [26], [27]. Not owning a helmet is consistently cited as one of the most common reasons for not using one, which led to the recommendation to include helmet provision in intervention programmes [28]. The probability of helmet use was increased four-fold among participants in the helmet and information group, and seven-fold among those of the helmet only group, compared with controls, suggesting at the very least no effect of information. The weakness of the information-based approach might be due to several factors. First, our data showed that the helmet was already massively recognised as an effective device to prevent head injuries. Giving additional information on the topic was thus hardly likely to change attitudes further. Second, our results are consistent with the finding that perception of one's own susceptibility to bicycle crashes is a major predictor of health behaviour and has a pivotal role in value-expectancy models such as the Health Belief Model [16]. The likelihood of bicycle-related injury was, however, perceived as moderate by our participants (Table 1), and those in the “information only” group were not provided with messages related to the magnitude of the risk. Altogether, provision of a helmet without any further messages is at least as effective as providing a helmet together with encouragement to wear it. This finding lends support to recent interest in so-called “nudging”, an approach that focuses on altering environmental cues rather than providing information to prompt healthier behaviour [29].

Results from determinant analysis (Table 4) showed that few components of the TPB were associated with observed helmet use. Most participants acknowledged helmet utility in preventing head injury (rated 8–10 on a 10-point scale by 80% of participants), but this perception had no influence on helmet use. Conversely, the belief that a helmet is useful in preventing facial injury was less consensual, but more influential. These results have consequences in terms of defining where to focus prevention messages. Head injury has so far received most attention because it is the leading cause of death and disability among cyclists. Facial injury is, however, also common among injured cyclists and might require surgery [30]. In addition, visible lesions resulting from facial injury have the potential to affect quality of life, self-image and social relationships [31], [32]. Our study therefore suggests the value of promoting the helmet as a face-protecting device. This may also influence design, since helmet use is associated with reductions in upper/mid facial injuries but not all facial injuries [3]. Pressure, especially from close family members and parents, has also been reported as a determinant of helmet use [33]. However, participants in our study reported little encouragement to wear a helmet from close family members (Table 1). Parental education toward injury prevention might be of value in increasing helmet use among children, thus modifying long-time habits that constitute a significant barrier to adoption of such behaviour [26].

### Limitations

The present study has limitations, mostly related to data collection in real-life situations. Only one-third of recruited participants (587 out of 1557) were observed by cameras and included in analyses. Even though sociodemographic characteristics, education, occupation and history of bicycle incidents did not differ between observed and unobserved participants, it cannot be excluded that unobserved participants may have had different behaviours regarding helmet use. In addition, even if they were not told that this was a study of helmet wearing, the fact that they were asked several questions about helmets might have influenced their behaviour. This influence, however, proved insufficient given the low rate of helmet use in the control group and given the fact that those randomised to the group with both helmet and information were less likely to use the helmet than those randomised to the helmet only group. Another concern was that while the sizes of the four randomisation groups were equivalent, this was not case for the number of observations. This was explained by a small number of outlier participants who were very frequently observed. Restricting the number of observations to 40 per participant substantially reduced the differences between groups without modifying OR estimates.

### Generalizability

Another point to consider is that this was a single-centre study, conducted in a French city with a high rate of bicycle use and among people with an urban way of living. Conclusions drawn from the study should therefore be applied to other settings with caution.

In conclusion, this study indicates that providing a helmet may be of value, but will not be sufficient to achieve high rates of helmet wearing among adult cyclists. Integrated and repeated prevention programmes that include free provision of helmets, parental education and communication on face protection might convince non-users to adopt this behaviour.

## References

[1] Constant A, Lagarde E (2010). Protecting vulnerable road users from injury.. PLoS Med.

[2] Liu BC, Ivers R, Norton R, Boufous S, Blows S (2008). Helmets for preventing injury in motorcycle riders.. Cochrane Database Syst Rev.

[3] Thompson DC, Rivara FP, Thompson R (2000). Helmets for preventing head and facial injuries in bicyclists.. Cochrane Database Syst Rev.

[4] ETSC (2003). Transport safety performance in the EU. A statistical Overview.

[5] Elvik R (2011). Publication bias and time-trend bias in meta-analysis of bicycle helmet efficacy: a re-analysis of Attewell, Glase and McFadden, 2001.. Accid Anal Prev.

[6] Attewell RG, Glase K, McFadden M (2001). Bicycle helmet efficacy: a meta-analysis.. Accid Anal Prev.

[7] Walker I (2007). Drivers overtaking bicyclists: objective data on the effects of riding position, helmet use, vehicle type and apparent gender.. Accid Anal Prev.

[8] Adams J, Hillman M (2001). The risk compensation theory and bicycle helmets.. Inj Prev.

[9] Robinson DL (2007). Bicycle helmet legislation: can we reach a consensus?. Accid Anal Prev.

[10] Robinson DL (1996). Head injuries and bicycle helmet laws.. Accid Anal Prev.

[11] Carnall D (1999). Cycle helmets should not be compulsory.. BMJ.

[12] Jacobsen PL (2003). Safety in numbers: more walkers and bicyclists, safer walking and bicycling.. Inj Prev.

[13] Royal ST, Kendrick D, Coleman T (2005). Non-legislative interventions for the promotion of cycle helmet wearing by children.. Cochrane Database Syst Rev.

[14] Ajzen I, Beckmann JKaJ (1985). From intentions to actions: a theory of planned behaviour.. Action control: From cognition to behaviour.

[15] Ajzen I (1988). Attitudes, personality and behaviour.

[16] Cummings KM, Jette AM, Rosenstock IM (1978). Construct validation of the health belief model.. Health Educ Monogr.

[17] Lajunen T, Rasanen M (2004). Can social psychological models be used to promote bicycle helmet use among teenagers? A comparison of the Health Belief Model, Theory of Planned Behavior and the Locus of Control.. J Safety Res.

[18] Jacques LB (1994). Rates of bicycle helmet use in an affluent Michigan County.. Public Health Rep.

[19] Kristiansen CM, Harding CM (1984). The social desirability of preventive health behavior.. Public Health Rep.

[20] Wiechman SA, Smith RE, Smoll FL, Ptacek JT (2000). Masking effects of social desirability response set on relations between psychosocial factors and sport injuries: a methodological note.. J Sci Med Sport.

[21] Chouinard E, Walter S (1995). Recall bias in case-control studies: an empirical analysis and theoretical framework.. J Clin Epidemiol.

[22] Zeger SL, Liang KY, Albert PS (1988). Models for longitudinal data: a generalized estimating equation approach.. Biometrics.

[23] Zeger SL, Liang KY (1986). Longitudinal data analysis for discrete and continuous outcomes.. Biometrics.

[24] Hanley JA, Negassa A, Edwardes MD, Forrester JE (2003). Statistical analysis of correlated data using generalized estimating equations: an orientation.. Am J Epidemiol.

[25] Villamor E, Hammer S, Martinez-Olaizola A (2008). Barriers to bicycle helmet use among Dutch paediatricians.. Child Care Health Dev.

[26] Forjuoh SN, Schuchmann JA, Fiesinger T, Mason S (2003). Parent-child concordance on reported barriers to helmet use by children.. Med Sci Monit.

[27] Thompson NJ, Sleet D, Sacks JJ (2002). Increasing the use of bicycle helmets: lessons from behavioral science.. Patient Educ Couns.

[28] OCDE/OECD (1998). Safety of vulnerable road users.

[29] Marteau TM, Ogilvie D, Roland M, Suhrcke M, Kelly MP (2011). Judging nudging: can nudging improve population health?. BMJ.

[30] Rivara FP, Thompson DC, Thompson RS (1997). Epidemiology of bicycle injuries and risk factors for serious injury.. Inj Prev.

[31] Rusch MD, Grunert BK, Sanger JR, Dzwierzynski WW, Matloub HS (2000). Psychological adjustment in children after traumatic disfiguring injuries: a 12-month follow-up.. Plast Reconstr Surg.

[32] Tebble NJ, Thomas DW, Price P (2004). Anxiety and self-consciousness in patients with minor facial lacerations.. J Adv Nurs.

[33] Caplow MP, Runyan CW (1995). Parental responses to a child bicycle helmet ordinance.. Am J Prev Med.

